# Supplementary material for: Hepatic insulin synthesis increases in rat models of diabetes mellitus type 1 and 2 differently
Source: PLoS One. 2023 Nov 29;18(11):e0294432. doi: 10.1371/journal.pone.0294432 (PMC10686419; doi:10.1371/journal.pone.0294432)
Supplement: S1 File — (DOCX) [file pone.0294432.s001.docx]

**S 1 Table.** **Fasting blood glucose (FBG).** Data are presented as mean ± standard error of mean (SEM); ND – non-diabetic group, T1D – type 1 diabetes, T2D – type 2 diabetes.

| **Number of rat** | **FBG, mmol/L** | | |
| --- | --- | --- | --- |
|  | ND | T1D | T2D |
| 1 | 5.9 | 10.6 | 9.3 |
| 2 | 5.2 | 12.7 | 8.5 |
| 3 | 5.4 | 9.1 | 8.7 |
| 4 | 6.5 | 11.1 | 10.5 |
| 5 | 3.8 | 10.9 | 10.3 |
| 6 | 6 | 10.9 | 8.9 |
| 7 | 5.9 | 8.5 | 9.2 |
| 8 | 3.9 | 8.9 | 15 |
| 9 | 4.3 | 10.7 | 14 |
| 10 | 5.1 | 8.7 | 10.4 |
|  |  |  |  |
| **Mean** | **5.20** | **10.21** | **10.48** |
| Standard deviation | 0.93 | 1.35 | 2.25 |
| **Standard error of the mean (SEM)** | **0.29** | **0.43** | **0.71** |

**S 2 Table.** **Glycosylated hemoglobin (HbA1c).** Data are presented as mean ± standard error of mean (SEM); ND – non-diabetic group, T1D – type 1 diabetes, T2D – type 2 diabetes.

| **Number of rat** | **HbA1c, %** | | |
| --- | --- | --- | --- |
|  | ND | T1D | T2D |
| 1 | 4.2 | 8.5 | 4.8 |
| 2 | 3.5 | 9 | 6.5 |
| 3 | 4.1 | 6.6 | 6.8 |
| 4 | 5.3 | 7.2 | 7.5 |
| 5 | 4.6 | 4.9 | 6.9 |
| 6 | 4.1 | 4.2 | 7.7 |
| 7 | 4.1 | 5.3 | 6.6 |
| 8 | 4.1 | 4.3 | 5.8 |
| 9 | 4.4 | 6.6 | 6.9 |
| 10 | 2.3 | 6 | 6.6 |
|  |  |  |  |
| **Mean** | **4.07** | **6.26** | **6.61** |
| Standard deviation | 0.77 | 1.65 | 0.83 |
| **Standard error of the mean (SEM)** | **0.24** | **0.52** | **0.26** |

**S 3 Table.** **Plasma insulin.** Data are presented as mean ± standard error of mean (SEM); ND – non-diabetic group, T1D – type 1 diabetes, T2D – type 2 diabetes.

| **Number of rat** | **Plasma insulin, mkg/L** | | |
| --- | --- | --- | --- |
|  | ND | T1D | T2D |
| 1 | 0.41 | 0.32 | 1 |
| 2 | 0.56 | 0.11 | 0.91 |
| 3 | 1.22 | 1.25 | 1 |
| 4 | 2.27 | 0.34 | 1.08 |
| 5 | 1.98 | 0.45 | 1.14 |
| 6 | 1.21 | 0.11 | 1.28 |
| 7 | 1.28 | 0.93 | 1.21 |
| 8 | 1.37 | 0.19 | 1.14 |
| 9 | 1.27 | 0.21 | 1.05 |
| 10 | 1.27 | 1.1 | 0.2 |
|  |  |  |  |
| **Mean** | **1.28** | **0.50** | **1.00** |
| Standard deviation | 0.55 | 0.43 | 0.30 |
| **Standard error of the mean** | **0.18** | **0.14** | **0.10** |

**S 4 Table.** **Homeostasis model assessment of insulin resistance (HOMA-IR).** Data are presented as mean ± standard error of mean (SEM); ND – non-diabetic group, T1D – type 1 diabetes, T2D – type 2 diabetes.

| **Number of rat** | **HOMA-IR** | | |
| --- | --- | --- | --- |
|  | ND | T1D | T2D |
| 1 | 2.51 | 3.95 | 9.71 |
| 2 | 3.06 | 4.46 | 8.52 |
| 3 | 6.9 | 11.9 | 11.98 |
| 4 | 14.44 | 3.35 | 11.86 |
| 5 | 7.87 | 5.73 | 13.32 |
| 6 | 7.57 | 4.25 | 11.95 |
| 7 | 7.22 | 8.27 | 11.61 |
| 8 | 5.6 | 1.73 | 18.95 |
| 9 | 5.7 | 4.35 | 15.34 |
| 10 | 6.16 | 10.01 | 2.76 |
|  |  |  |  |
| **Mean** | **6.70** | **5.80** | **11.60** |
| Standard deviation | 3.26 | 3.22 | 4.25 |
| **Standard error of the mean** | **1.03** | **1.02** | **1.34** |

**S 5 Table.** **Oral glucose tolerance test (OGTT) for non-diabetic rats.** Data are presented as mean ± standard error of mean (SEM); ND – non-diabetic group, T1D – type 1 diabetes, T2D – type 2 diabetes.

| **Number of rat** | **Glucose, mmol/L** | | | | | **Area under the curve (AUC)** |
| --- | --- | --- | --- | --- | --- | --- |
|  | **Fasting** | **After glucose intake (1 g/kg b.w.), min.** | | | |  |
|  |  | **30** | **60** | **90** | **120** |  |
| 1 | 3.9 | 8.7 | 6.8 | 6.3 | 4.9 | 786 |
| 2 | 5.7 | 8.3 | 8 | 5.3 | 5.6 | 817.5 |
| 3 | 5.4 | 9.5 | 5.3 | 5.9 | 5.2 | 780 |
| 4 | 5.2 | 8.9 | 7.2 | 6.1 | 5.9 | 832.5 |
| 5 | 4.8 | 8.7 | 6.9 | 6.2 | 5.7 | 811.5 |
| 6 | 5.2 | 8 | 5.8 | 5.5 | 4.5 | 724.5 |
|  |  |  |  |  |  |  |
| **Mean** | **5.03** | **8.68** | **6.67** | **5.88** | **5.30** | **792.00** |
| Standard deviation | 0.63 | 0.52 | 0.98 | 0.40 | 0.53 | 38.49 |
| **Standard error of the mean** | **0.26** | **0.21** | **0.40** | **0.16** | **0.22** | **15.71** |

**S 6 Table.** **Oral glucose tolerance test (OGTT) for T2D rats.** Data are presented as mean ± standard error of mean (SEM); ND – non-diabetic group, T1D – type 1 diabetes, T2D – type 2 diabetes.

| **Number of rat** | **Glucose, mmol/L** | | | | | **Area under the curve (AUC)** |
| --- | --- | --- | --- | --- | --- | --- |
|  | **Fasting** | **After glucose intake (1 g/kg b.w.), min.** | | | |  |
|  |  | **30** | **60** | **90** | **120** |  |
| 1 | 8.9 | 16.3 | 13.9 | 12.8 | 11.2 | 1591.5 |
| 2 | 9.2 | 15.9 | 12.8 | 11.8 | 11.6 | 1527 |
| 3 | 10.3 | 16.8 | 12 | 10.7 | 10.7 | 1500 |
| 4 | 8.8 | 15.7 | 13.2 | 11.8 | 10.7 | 1513.5 |
| 5 | 9.3 | 15.5 | 13 | 12.1 | 10.5 | 1515 |
| 6 | 10 | 16.2 | 12.7 | 11.7 | 11.3 | 1537.5 |
|  |  |  |  |  |  |  |
| **Mean** | **9.42** | **16.07** | **12.93** | **11.82** | **11.00** | **1530.75** |
| Standard deviation | 0.60 | 0.47 | 0.63 | 0.68 | 0.43 | 32.38 |
| **Standard error of the mean** | **0.25** | **0.19** | **0.26** | **0.28** | **0.18** | **13.22** |

**S 7 Table.** **Aspartate aminotransferase (AST) blood test.** Data are presented as mean ± standard error of mean (SEM); ND – non-diabetic group, T1D – type 1 diabetes, T2D – type 2 diabetes.

| **Number of rat** | **AST, mkmol/min*L** | | |
| --- | --- | --- | --- |
|  | **ND** | **T1D** | **T2D** |
| 1 | 12.5 | 18.9 | 16.46 |
| 2 | 14.5 | 18.23 | 22 |
| 3 | 13.3 | 18.4 | 17.2 |
| 4 | 19.9 | 23.3 | 15.2 |
| 5 | 19.3 | 21.6 | 16.78 |
| 6 | 18.4 | 23.5 | 16.5 |
| 7 | 14.4 | 19.7 | 17.8 |
| 8 | 18 | 17.2 | 15.45 |
| 9 | 17.2 | 20.4 | 16.3 |
| 10 | 18.2 | 21.5 | 15.2 |
|  |  |  |  |
| **Mean** | **16.57** | **20.27** | **16.89** |
| Standard deviation | 2.65 | 2.17 | 1.99 |
| **Standard error of the mean** | **0.84** | **0.68** | **0.63** |

**S 8 Table.** **Alanine aminotransferase (ALT) blood test.** Data are presented as mean ± standard error of mean (SEM); ND – non-diabetic group, T1D – type 1 diabetes, T2D – type 2 diabetes.

| **Number of rat** | **ALT, mkmol/min*L** | | |
| --- | --- | --- | --- |
|  | **ND** | **T1D** | **T2D** |
| 1 | 12.3 | 18.9 | 18.4 |
| 2 | 14.1 | 12.7 | 22.5 |
| 3 | 14.1 | 16.2 | 24.17 |
| 4 | 14.8 | 17.4 | 14.5 |
| 5 | 9.3 | 20.1 | 17.6 |
| 6 | 9.5 | 16.3 | 18 |
| 7 | 11 | 17 | 24.2 |
| 8 | 14.8 | 12.9 | 27.1 |
| 9 | 14.6 | 14 | 22.5 |
| 10 | 14.4 | 16 | 21.9 |
|  |  |  |  |
| **Mean** | **12.89** | **16.15** | **21.09** |
| Standard deviation | 2.20 | 2.42 | 3.83 |
| **Standard error of the mean** | **0.70** | **0.76** | **1.21** |

**S 9 Table.** **AST/ALT ratio.** Data are presented as mean ± standard error of mean (SEM); ND – non-diabetic group, T1D – type 1 diabetes, T2D – type 2 diabetes.

| **Number of rat** | **AST/ALT** | | |
| --- | --- | --- | --- |
|  | **ND** | **T1D** | **T2D** |
| 1 | 1.25 | 1.34 | 1.1 |
| 2 | 1.23 | 1.07 | 1.16 |
| 3 | 1.24 | 1.44 | 0.84 |
| 4 | 1.73 | 1.16 | 1.05 |
| 5 | 1.26 | 1.33 | 0.74 |
| 6 | 1.52 | 1.46 | 0.92 |
| 7 | 1.04 | 1 | 0.74 |
| 8 | 1.22 | 1.18 | 0.5 |
| 9 | 1.18 | 0.99 | 0.77 |
| 10 | 1.26 | 1.77 | 0.72 |
|  |  |  |  |
| **Mean** | **1.29** | **1.27** | **0.85** |
| Standard deviation | 0.19 | 0.24 | 0.20 |
| **Standard error of the mean** | **0.06** | **0.08** | **0.06** |

**S 10 Table.** **Alkaline phosphatase (ALP) blood test.** Data are presented as mean ± standard error of mean (SEM); ND – non-diabetic group, T1D – type 1 diabetes, T2D – type 2 diabetes.

| **Number of rat** | **ALP, mkmol/min*L** | | |
| --- | --- | --- | --- |
|  | **ND** | **T1D** | **T2D** |
| 1 | 59.8 | 49.2 | 58.9 |
| 2 | 78.9 | 81.4 | 56 |
| 3 | 63 | 35.6 | 58.1 |
| 4 | 79.3 | 51.9 | 69 |
| 5 | 81.7 | 53.1 | 80.4 |
| 6 | 51.9 | 109.1 | 87.4 |
| 7 | 69.1 | 59.3 | 44.2 |
| 8 | 51.8 | 60 | 64.9 |
| 9 | 79.4 | 62.6 | 53.1 |
| 10 | 81.7 | 41.5 | 67.2 |
|  |  |  |  |
| **Mean** | **69.66** | **60.37** | **63.92** |
| Standard deviation | 12.20 | 21.19 | 12.86 |
| **Standard error of the mean** | **3.86** | **6.70** | **4.07** |

**S 11 Table.** **Total protein in blood.** Data are presented as mean ± standard error of mean (SEM); ND – non-diabetic group, T1D – type 1 diabetes, T2D – type 2 diabetes.

| **Number of rat** | **Total protein, g/L** | | |
| --- | --- | --- | --- |
|  | **ND** | **T1D** | **T2D** |
| 1 | 71.7 | 59.4 | 66.6 |
| 2 | 66 | 62.7 | 59.4 |
| 3 | 70.8 | 61.8 | 67 |
| 4 | 75.5 | 56.1 | 54.1 |
| 5 | 67.5 | 61.8 | 56.3 |
| 6 | 72.3 | 52.7 | 68.4 |
| 7 | 66.5 | 56.4 | 70.8 |
| 8 | 69.8 | 51.9 | 65.6 |
| 9 | 72.91 | 55.2 | 68.4 |
| 10 | 73.96 | 62.2 | 70.8 |
|  |  |  |  |
| **Mean** | **70.70** | **58.02** | **64.74** |
| Standard deviation | 3.21 | 4.08 | 5.99 |
| **Standard error of the mean** | **1.02** | **1.29** | **1.89** |

**S 12 Table.** **White blood cells (WBC) count.** Data are presented as mean ± standard error of mean (SEM); ND – non-diabetic group, T1D – type 1 diabetes, T2D – type 2 diabetes.

| **Number of rat** | **WBC, 10^9^/L** | | |
| --- | --- | --- | --- |
|  | **ND** | **T1D** | **T2D** |
| 1 | 7.23 | 10.5 | 14.3 |
| 2 | 7.5 | 11 | 14.2 |
| 3 | 7.01 | 11.10 | 18.1 |
| 4 | 6.7 | 8.6 | 17.1 |
| 5 | 9.31 | 11.6 | 17.9 |
| 6 | 9.42 | 10.41 | 17.7 |
| 7 | 6.92 | 12.86 | 11.9 |
| 8 | 6.8 | 12.26 | 18.2 |
| 9 | 9 | 11.82 | 9.4 |
| 10 | 7.8 | 12.91 | 6.6 |
|  |  |  |  |
| **Mean** | **7.77** | **11.31** | **14.54** |
| Standard deviation | 1.07 | 1.30 | 4.09 |
| **Standard error of the mean** | **0.34** | **0.41** | **1.29** |

**S 13 Table.** **Lymphocytes (LYM) count.** Data are presented as mean ± standard error of mean (SEM); ND – non-diabetic group, T1D – type 1 diabetes, T2D – type 2 diabetes.

| **Number of rat** | **LYM, 10^9^/L** | | |
| --- | --- | --- | --- |
|  | **ND** | **T1D** | **T2D** |
| 1 | 4.4 | 5.6 | 10.4 |
| 2 | 3.5 | 6.6 | 9.7 |
| 3 | 4.6 | 6 | 12.5 |
| 4 | 3.4 | 4.4 | 11.6 |
| 5 | 4 | 5.5 | 12.4 |
| 6 | 5.5 | 5.39 | 10.3 |
| 7 | 3.9 | 6.89 | 10.3 |
| 8 | 4.4 | 5.5 | 4.8 |
| 9 | 3.2 | 6.20 | 4.1 |
| 10 | 6.1 | 7.2 | 7.2 |
|  |  |  |  |
| **Mean** | **4.30** | **5.93** | **9.33** |
| Standard deviation | 0.92 | 0.83 | 2.99 |
| **Standard error of the mean** | **0.29** | **0.26** | **0.94** |

**S 14 Table.** **Granulocytes (GRA) count.** Data are presented as mean ± standard error of mean (SEM); ND – non-diabetic group, T1D – type 1 diabetes, T2D – type 2 diabetes.

| **Number of rat** | **GRA, 10^9^/L** | | |
| --- | --- | --- | --- |
|  | **ND** | **T1D** | **T2D** |
| 1 | 2.85 | 3.7 | 2.3 |
| 2 | 2.2 | 4.6 | 2.8 |
| 3 | 1.2 | 4 | 3.8 |
| 4 | 3.1 | 3 | 3.8 |
| 5 | 2.1 | 4.8 | 3.4 |
| 6 | 1.9 | 4.45 | 5.5 |
| 7 | 2.99 | 4.72 | 5.7 |
| 8 | 2.7 | 4.73 | 4.3 |
| 9 | 3.1 | 4.72 | 1.8 |
| 10 | 0.2 | 4.06 | 4.3 |
|  |  |  |  |
| **Mean** | **2.23** | **4.28** | **3.77** |
| Standard deviation | 0.94 | 0.59 | 1.26 |
| **Standard error of the mean** | **0.30** | **0.19** | **0.40** |

**S 15 Table.** **Mitotic index in liver cells.** Data are presented as mean ± standard error of mean (SEM); ND – non-diabetic group, T1D – type 1 diabetes, T2D – type 2 diabetes.

| **Number of rat** | **Mitotic index, %** | | |
| --- | --- | --- | --- |
|  | **ND** | **T1D** | **T2D** |
| 1 | 31.45 | 43.79 | 56.91 |
| 2 | 24.39 | 51.65 | 48.19 |
| 3 | 24.92 | 49.97 | 63.71 |
| 4 | 29.31 | 47.95 | 78.91 |
| 5 | 26.22 | 44.99 | 56.80 |
| 6 | 30.16 | 51.73 | 52.14 |
| 7 | 26.89 | 45.17 | 55.67 |
| 8 | 30.07 | 45.37 | 47.14 |
| 9 | 28.88 | 49.13 | 54.56 |
| 10 | 28.14 | 48.33 | 53.28 |
|  |  |  |  |
| **Mean** | **28.04** | **47.81** | **56.73** |
| Standard deviation | 2.36 | 2.86 | 9.10 |
| **Standard error of the mean** | **0.75** | **0.91** | **2.88** |

**S 16 Table.** **Number of binucleated hepatocytes.** Data are presented as mean ± standard error of mean (SEM); ND – non-diabetic group, T1D – type 1 diabetes, T2D – type 2 diabetes.

| **Number of rat** | **Binucleated hepatocytes, N/mm^2^** | | |
| --- | --- | --- | --- |
|  | **ND** | **T1D** | **T2D** |
| 1 | 28.10 | 49.06 | 33.04 |
| 2 | 24.39 | 47.88 | 45.78 |
| 3 | 29.90 | 49.92 | 44.02 |
| 4 | 24.91 | 52.91 | 38.93 |
| 5 | 29.41 | 47.82 | 29.72 |
| 6 | 24.24 | 52.25 | 38.02 |
| 7 | 28.71 | 47.04 | 36.65 |
| 8 | 26.23 | 48.96 | 37.16 |
| 9 | 24.44 | 50.75 | 38.89 |
| 10 | 27.01 | 52.88 | 37.14 |
|  |  |  |  |
| **Mean** | **26.73** | **49.95** | **37.93** |
| Standard deviation | 2.20 | 2.17 | 4.65 |
| **Standard error of the mean** | **0.70** | **0.69** | **1.47** |

**S 17 Table.** **Number of liver sinusoidal cells.** Data are presented as mean ± standard error of mean (SEM); ND – non-diabetic group, T1D – type 1 diabetes, T2D – type 2 diabetes.

| **Number of rat** | **Sinusoidal cells, N/mm^2^** | | |
| --- | --- | --- | --- |
|  | **ND** | **T1D** | **T2D** |
| 1 | 369.72 | 765.18 | 475.19 |
| 2 | 402.52 | 559.12 | 455.21 |
| 3 | 379.97 | 648.32 | 874.36 |
| 4 | 386.54 | 704.22 | 870.11 |
| 5 | 397.03 | 549.38 | 891.06 |
| 6 | 382.15 | 611.99 | 789.47 |
| 7 | 399.02 | 702.17 | 676.87 |
| 8 | 385.38 | 611.67 | 526.18 |
| 9 | 378.26 | 639.21 | 763.17 |
| 10 | 394.98 | 661.12 | 810.29 |
|  |  |  |  |
| Mean | **387.56** | **645.24** | **713.19** |
| Standard deviation | 10.53 | 66.87 | 169.79 |
| Standard error of the mean | **3.24** | **8.18** | **13.03** |

**S 18 Table.** **Number of macrophages in liver tissue.** Data are presented as mean ± standard error of mean (SEM); ND – non-diabetic group, T1D – type 1 diabetes, T2D – type 2 diabetes.

| **Number of rat** | **Macrophages, N/mm^2^** | | |
| --- | --- | --- | --- |
|  | **ND** | **T1D** | **T2D** |
| 1 | 110.87 | 241.97 | 142.5 |
| 2 | 120.71 | 176.73 | 136.51 |
| 3 | 113.95 | 204.93 | 262.22 |
| 4 | 115.92 | 222.69 | 260.94 |
| 5 | 119.06 | 173.95 | 267.52 |
| 6 | 112.89 | 210.38 | 209.78 |
| 7 | 118.78 | 189.17 | 214.89 |
| 8 | 116.99 | 204.16 | 236.89 |
| 9 | 114.12 | 215.47 | 183.58 |
| 10 | 117.68 | 201.05 | 224.58 |
|  |  |  |  |
| Mean | **116.10** | **204.05** | **213.94** |
| Standard deviation | 3.10 | 20.65 | 47.25 |
| Standard error of the mean | **1.76** | **4.54** | **6.87** |

**S 19 Table.** **Total number of CD45+ cells in liver.** Data are presented as mean ± standard error of mean (SEM); ND – non-diabetic group, T1D – type 1 diabetes, T2D – type 2 diabetes.

| **Number of rat** | **CD45+ cells, N/mm^2^** | | |
| --- | --- | --- | --- |
|  | **ND** | **T1D** | **T2D** |
| 1 | 31.02 | 89.97 | 89.7 |
| 2 | 14.18 | 66.3 | 70.2 |
| 3 | 31.18 | 62.4 | 105.3 |
| 4 | 30.11 | 72.76 | 58.5 |
| 5 | 29.33 | 69.56 | 46.8 |
| 6 | 27.17 | 72.86 | 72.18 |
| 7 | 31.18 | 63.22 | 61.19 |
| 8 | 21.25 | 79.96 | 84.17 |
| 9 | 30.22 | 81.74 | 67.36 |
| 10 | 25.41 | 69.97 | 86.16 |
|  |  |  |  |
| **Mean** | **27.11** | **72.87** | **74.16** |
| Standard deviation | 5.54 | 8.73 | 17.27 |
| **Standard error of the mean** | **1.75** | **2.76** | **5.46** |

**S 20 Table.** **Number of CD45+ cells in liver parenchyma.** Data are presented as mean ± standard error of mean (SEM); ND – non-diabetic group, T1D – type 1 diabetes, T2D – type 2 diabetes.

| **Number of rat** | **CD45+ cells in liver parenchyma, N/mm^2^** | | |
| --- | --- | --- | --- |
|  | **ND** | **T1D** | **T2D** |
| 1 | 9.83 | 50.67 | 44.1 |
| 2 | 7 | 35.1 | 35.1 |
| 3 | 14.9 | 42.9 | 39 |
| 4 | 16.6 | 51.97 | 35.1 |
| 5 | 10.82 | 43.78 | 31.2 |
| 6 | 12.96 | 45.97 | 48.97 |
| 7 | 8.86 | 48.67 | 46.87 |
| 8 | 15.82 | 49.84 | 43.64 |
| 9 | 9.67 | 46.87 | 50.71 |
| 10 | 11.86 | 52.96 | 47.89 |
|  |  |  |  |
| **Mean** | **11.83** | **46.87** | **42.26** |
| Standard deviation | 3.18 | 5.32 | 6.75 |
| **Standard error of the mean** | **1.01** | **1.68** | **2.13** |

**S 21 Table.** **Number of CD45+ cells in perivascular area in liver.** Data are presented as mean ± standard error of mean (SEM); ND – non-diabetic group, T1D – type 1 diabetes, T2D – type 2 diabetes.

| **Number of rat** | **Perivascular CD45+ cells, N/mm^2^** | | |
| --- | --- | --- | --- |
|  | **ND** | **T1D** | **T2D** |
| 1 | 21.19 | 27.03 | 15.6 |
| 2 | 7.18 | 31.2 | 35.1 |
| 3 | 16.28 | 19.5 | 66.31 |
| 4 | 13.51 | 26.14 | 23.4 |
| 5 | 18.51 | 22.66 | 15.6 |
| 6 | 17.84 | 30.33 | 24.16 |
| 7 | 14.96 | 26.43 | 36.67 |
| 8 | 12.65 | 29.14 | 33.92 |
| 9 | 17.34 | 21.16 | 27.89 |
| 10 | 13.86 | 25.47 | 34.12 |
|  |  |  |  |
| **Mean** | **15.33** | **25.91** | **31.28** |
| Standard deviation | 3.87 | 3.86 | 14.54 |
| **Standard error of the mean** | **1.22** | **1.22** | **4.60** |

**S 22 Table.** **Total number of CD3+ cells in liver.** Data are presented as mean ± standard error of mean (SEM); ND – non-diabetic group, T1D – type 1 diabetes, T2D – type 2 diabetes.

| **Number of rat** | **CD3+ cells, N/mm^2^** | | |
| --- | --- | --- | --- |
|  | **ND** | **T1D** | **T2D** |
| 1 | 5.6 | 33.78 | 30.98 |
| 2 | 5.2 | 22.97 | 35.13 |
| 3 | 5.15 | 39.88 | 47.1 |
| 4 | 3 | 15.59 | 44.94 |
| 5 | 7.05 | 21.01 | 50.16 |
| 6 | 5.41 | 27.56 | 47.18 |
| 7 | 7.01 | 20.74 | 46.92 |
| 8 | 5.23 | 35.87 | 41.18 |
| 9 | 3.02 | 23.76 | 39.96 |
| 10 | 5.12 | 25.86 | 45.19 |
|  |  |  |  |
| **Mean** | **5.18** | **26.70** | **42.87** |
| Standard deviation | 1.35 | 7.62 | 6.04 |
| **Standard error of the mean** | **0.43** | **2.41** | **1.91** |

**S 23 Table.** **Number of CD3+ cells in liver parenchyma.** Data are presented as mean ± standard error of mean (SEM); ND – non-diabetic group, T1D – type 1 diabetes, T2D – type 2 diabetes.

| **Number of rat** | **CD3+ cells in liver parenchyma, N/mm^2^** | | |
| --- | --- | --- | --- |
|  | **ND** | **T1D** | **T2D** |
| 1 | 2 | 15.55 | 13.91 |
| 2 | 1.2 | 9.8 | 19.04 |
| 3 | 1.08 | 18.78 | 18.01 |
| 4 | 1 | 9.51 | 23.69 |
| 5 | 1.27 | 7.99 | 22.85 |
| 6 | 1.24 | 14.96 | 21.78 |
| 7 | 1.71 | 10.76 | 17.96 |
| 8 | 1.34 | 9.19 | 19.14 |
| 9 | 1.09 | 16.78 | 18.75 |
| 10 | 1.16 | 9.96 | 20.12 |
|  |  |  |  |
| **Mean** | **1.31** | **12.33** | **19.53** |
| Standard deviation | 0.31 | 3.80 | 2.81 |
| **Standard error of the mean** | **0.10** | **1.20** | **0.89** |

**S 24 Table.** **Number of CD3+ cells in perivascular area in liver.** Data are presented as mean ± standard error of mean (SEM); ND – non-diabetic group, T1D – type 1 diabetes, T2D – type 2 diabetes.

| **Number of rat** | **Perivascular CD3+ cells, N/mm^2^** | | |
| --- | --- | --- | --- |
|  | **ND** | **T1D** | **T2D** |
| 1 | 3.6 | 18.23 | 13.27 |
| 2 | 4 | 13.17 | 16.09 |
| 3 | 4.07 | 21.1 | 39.09 |
| 4 | 2 | 6.08 | 21.26 |
| 5 | 5.78 | 13.02 | 27.31 |
| 6 | 4.56 | 12.86 | 19.46 |
| 7 | 3.67 | 17.72 | 23.24 |
| 8 | 3.31 | 15.62 | 28.16 |
| 9 | 4.23 | 11.52 | 29.16 |
| 10 | 3.74 | 13.86 | 17.17 |
|  |  |  |  |
| **Mean** | **3.90** | **14.32** | **23.42** |
| Standard deviation | 0.96 | 4.16 | 7.69 |
| **Standard error of the mean** | **0.30** | **1.31** | **2.43** |

**S 25 Table.** **Number of Pdx1+ cells in liver.** Data are presented as mean ± standard error of mean (SEM); ND – non-diabetic group, T1D – type 1 diabetes, T2D – type 2 diabetes.

| **Number of rat** | **Pdx1+ cells, N/mm^2^** | | |
| --- | --- | --- | --- |
|  | **ND** | **T1D** | **T2D** |
| 1 | 28.4 | 46.06 | 16.68 |
| 2 | 35.82 | 43.32 | 31.53 |
| 3 | 21.76 | 39 | 28.43 |
| 4 | 37.21 | 43.31 | 69.03 |
| 5 | 37.42 | 41.91 | 25.04 |
| 6 | 25.11 | 46.72 | 20.76 |
| 7 | 34.12 | 43.23 | 45.31 |
| 8 | 35.13 | 45.56 | 18.76 |
| 9 | 37.09 | 43.82 | 36.28 |
| 10 | 29.12 | 47.33 | 29.73 |
|  |  |  |  |
| **Mean** | **32.12** | **44.03** | **32.16** |
| Standard deviation | 5.62 | 2.50 | 15.53 |
| **Standard error of the mean** | **1.78** | **0.79** | **4.91** |

**S 26 Table.** **Number of MafA+ cells in liver.** Data are presented as mean ± standard error of mean (SEM); ND – non-diabetic group, T1D – type 1 diabetes, T2D – type 2 diabetes.

| **Number of rat** | **MafA+ cells, N/mm^2^** | | |
| --- | --- | --- | --- |
|  | **ND** | **T1D** | **T2D** |
| 1 | 17.3 | 43.53 | 33.26 |
| 2 | 16.11 | 36.94 | 22.05 |
| 3 | 18.2 | 23.83 | 20.68 |
| 4 | 16.1 | 29.97 | 25.16 |
| 5 | 19 | 38.69 | 27.14 |
| 6 | 17.15 | 36.37 | 26.14 |
| 7 | 16.68 | 27.14 | 24.87 |
| 8 | 18.24 | 40.17 | 22.19 |
| 9 | 18.22 | 42.12 | 21.53 |
| 10 | 16.43 | 39.09 | 23.62 |
|  |  |  |  |
| **Mean** | **17.34** | **35.79** | **24.66** |
| Standard deviation | 1.02 | 6.60 | 3.69 |
| **Standard error of the mean** | **0.32** | **2.09** | **1.17** |

**S 27 Table.** **Number of Ngn3+ cells in liver.** Data are presented as mean ± standard error of mean (SEM); ND – non-diabetic group, T1D – type 1 diabetes, T2D – type 2 diabetes.

| **Number of rat** | **Ngn3+ cells, N/mm^2^** | | |
| --- | --- | --- | --- |
|  | **ND** | **T1D** | **T2D** |
| 1 | 75.18 | 69.77 | 82.26 |
| 2 | 106.83 | 76.95 | 77.72 |
| 3 | 89.29 | 108.67 | 122.55 |
| 4 | 115.77 | 118.68 | 87.73 |
| 5 | 68.09 | 78.92 | 89.34 |
| 6 | 83.16 | 96.16 | 91.23 |
| 7 | 100.14 | 102.84 | 117.81 |
| 8 | 93.17 | 110.26 | 83.16 |
| 9 | 97.55 | 83.28 | 94.55 |
| 10 | 81.12 | 79.31 | 85.77 |
|  |  |  |  |
| **Mean** | **91.03** | **92.48** | **93.21** |
| Standard deviation | 14.65 | 16.95 | 15.03 |
| **Standard error of the mean** | **4.63** | **5.36** | **4.75** |

**S 28 Table.** **Number of KRT19+ cells in liver.** Data are presented as mean ± standard error of mean (SEM); ND – non-diabetic group, T1D – type 1 diabetes, T2D – type 2 diabetes.

| **Number of rat** | **KRT19+ cells, N/mm^2^** | | |
| --- | --- | --- | --- |
|  | **ND** | **T1D** | **T2D** |
| 1 | 47.42 | 71.14 | 71.14 |
| 2 | 57.41 | 43.61 | 48.67 |
| 3 | 45.13 | 54.87 | 71.14 |
| 4 | 48.19 | 49.12 | 56.29 |
| 5 | 43.93 | 68.33 | 71.05 |
| 6 | 52.76 | 62.08 | 71.66 |
| 7 | 54.52 | 50.26 | 70.82 |
| 8 | 55.45 | 67.19 | 52.43 |
| 9 | 53.12 | 55.38 | 54.24 |
| 10 | 52.27 | 52.16 | 69.14 |
|  |  |  |  |
| **Mean** | **51.02** | **57.41** | **63.66** |
| Standard deviation | 4.57 | 9.27 | 9.46 |
| **Standard error of the mean** | **1.44** | **2.93** | **2.99** |

**S 29 Table.** **Number of OV6+ cells in liver.** Data are presented as mean ± standard error of mean (SEM); ND – non-diabetic group, T1D – type 1 diabetes, T2D – type 2 diabetes.

| **Number of rat** | **OV6+ cells, N/mm^2^** | | |
| --- | --- | --- | --- |
|  | **ND** | **T1D** | **T2D** |
| 1 | 103.72 | 129.86 | 120.64 |
| 2 | 108.54 | 140.61 | 152.26 |
| 3 | 87.79 | 152.26 | 136.58 |
| 4 | 88.76 | 156.72 | 155.45 |
| 5 | 90.72 | 108.45 | 118.01 |
| 6 | 88.23 | 131.22 | 146.77 |
| 7 | 83.88 | 129.46 | 130.94 |
| 8 | 92.93 | 123.17 | 125.85 |
| 9 | 93.57 | 115.82 | 131.68 |
| 10 | 90.99 | 118.26 | 147.73 |
|  |  |  |  |
| **Mean** | **92.91** | **130.58** | **136.59** |
| Standard deviation | 7.58 | 15.53 | 13.33 |
| **Standard error of the mean** | **2.40** | **4.91** | **4.22** |

**S 30 Table.** **Number of insulin+ hepatocytes.** Data are presented as mean ± standard error of mean (SEM); ND – non-diabetic group, T1D – type 1 diabetes, T2D – type 2 diabetes.

| **Number of rat** | **Insulin+ hepatocytes, N/mm^2^** | | |
| --- | --- | --- | --- |
|  | **ND** | **T1D** | **T2D** |
| 1 | 7.18 | 23.09 | 125.20 |
| 2 | 19.30 | 37.18 | 193.90 |
| 3 | 23.57 | 17.02 | 145.87 |
| 4 | 8.19 | 31.98 | 99.73 |
| 5 | 13.33 | 15.21 | 192.64 |
| 6 | 11.23 | 30.48 | 175.14 |
| 7 | 18.69 | 33.68 | 133.59 |
| 8 | 16.96 | 21.86 | 126.76 |
| 9 | 9.84 | 38.11 | 111.21 |
| 10 | 13.78 | 16.94 | 137.66 |
|  |  |  |  |
| **Mean** | **14.21** | **26.56** | **144.17** |
| Standard deviation | 5.330712 | 8.744098 | 32.76686 |
| **Standard error of the mean** | **1.68572** | **2.76513** | **10.3618** |

**S 31 Table.** **Number of insulin+ sinusoidal cells in liver.** Data are presented as mean ± standard error of mean (SEM); ND – non-diabetic group, T1D – type 1 diabetes, T2D – type 2 diabetes.

| **Number of rat** | **Insulin+ sinusoidal cells, N/mm^2^** | | |
| --- | --- | --- | --- |
|  | **ND** | **T1D** | **T2D** |
| 1 | **ND** | **T1D** | **T2D** |
| 2 | 12.22 | 18.92 | 44.90 |
| 3 | 18.82 | 26.17 | 10.29 |
| 4 | 12.53 | 21.13 | 20.10 |
| 5 | 10.07 | 15.59 | 43.81 |
| 6 | 15.55 | 25.96 | 69.90 |
| 7 | 14.29 | 17.49 | 35.25 |
| 8 | 11.57 | 25.11 | 49.17 |
| 9 | 17.11 | 21.51 | 25.39 |
| 10 | 12.09 | 16.94 | 39.41 |
|  | 13.88 | 19.42 | 52.19 |
| **Mean** |  |  |  |
| Standard deviation | **13.81** | **20.82** | **39.04** |
| **Standard error of the mean** | 2.695325 | 3.845408 | 17.2275 |
