# Supplementary material for: Hepatic insulin synthesis increases in rat models of diabetes mellitus type 1 and 2 differently
Source: PLoS One. 2023 Nov 29;18(11):e0294432. doi: 10.1371/journal.pone.0294432 (PMC10686419; doi:10.1371/journal.pone.0294432)
Supplement: Attachment — Submitted filename: Reviwer Recommendation.pdf [file pone.0294432.s002.pdf]

Reviewer Recommendation

The manuscript "Hepatic Insulin Synthesis Increases in Rat Models of Diabetes Mellitus Type 1 and 2 Differently" investigates insulin positive cells in the liver by different methods in both types of diabetes mellitus which is interesting; however, I think this manuscript and its figures need major revision.

General comments:

- Manuscript needs English language revising
- Paragraph should not start with abbreviation, so please write the full name, and here are some examples: IPS "third paragraph in introduction", T1D "second paragraph in material and methods", …ect.

Abstract:

- Line 6; Replace were not (done) with were not (performed).

Introduction:

- Correct the following words: per-manent to **permanent**, idi-opathic, proinsulin-, ex-pression, in-sulin, vec-tor, peculiar-ities, insu-lin, transdif-ferentiate, insu-lin, phago-, immunolog-ical, pit-cells, inflammation – in,
- Introduction is too long, there is no clear coherence and sequence in the writing, and most of the paragraphs require reconstruction and here are some examples for improvement:

Old one: In any case, the number of the functioning β-cells is insufficient in both T1D and T2D, and β-cells loss and dysfunction are the main reasons of hyperglycemia and associated with it complications [5].
New one: Loss and dysfunction of β-cells are the main reasons of hyperglycemia and its associated complications in both types of DM [5].

Old one: first, they are an additional source of insulin, the increase in the significance of which for the organism is logical to assume when pancreatic islets β-cells are damaged. Earlier we demonstrated [8] that in experimental T2D mass and functional activity of islet β-cells decrease faster, than of IPCs in pancreatic acini and ducts. Understanding the mechanisms and conditions, leading to the generation of IPCs in organs, are able to alleviate the problem of lack of β-cells. Second, since knowledge about ectopic insulin expression and, more, developmental plasticity of adult somatic cells and its reprogramming to committed lineages, unsufficient for complete and clear picture of this phenomenon, studying of extra-pancreatic insulin expression is also of theoretical interest.
New one: first, they are additional sources of insulin, as they showed significant increase within organism that are able to alleviate the problem when pancreatic islets β-cells are damaged as recorded in our previous study [8]. Second, to disclose the extra pancreatic insulin expression from adult somatic cells and reprogramming it to committed lineages.

- Write the full name of the Pdx1 abbreviation within the text.
- Rewrite the aim of the study in a simple and clear form.

Material and methods:

- Correct the following words: ac-cording, fluo-rescent.
- Insulin+, macrophages (F4/80+ cells), oval cells (OV6+ and KRT19+ cells), CD3+ and CD45+ cells were detected by IHC, **(replace** and **by** while**)** Pdx1+, MafA+, Ngn3+ cells were detected using fluorescent IHC.
- Add data about the color and site of immune-expression (cytoplasmic or nuclear) in the stained cells.
- Morphometric analysis: specify the number of captured field from each slide/group.

RESULTS

Result section needs SUBSTANTIAL revision.

- Correct the following words: ob-served, insu-lin, De-pending, ana-lyzed, pro-tein, particu-larly, rear-rangement, regenera-tive,
- Write the full name of the following abbreviations within the text (AUC, HOMA-IR).
- Figure (1 C) compares ND and T2D, while figure legend compares T2D from T1D, revise it.
- Results of (Figure 1 from F to J) are not mentioned in figure legend, need to be added.
- Results of (Figure 2 A, C to I) are not mentioned in the text, need to be added.
- Add scale bar in all photomicrographs in figure 2.
- Reorganize the writing of result section (text and legend) according to figures distribution. For example authors write about figure 1 A-D, then fig 2B, then figure 6, then return to figure 1F-J and so on…...
- For scientific benefit, it is better to insert each bar charts immediately below their corresponding histological or IHC figures.
- Figures 5 and 6 are the same figure???? Need to insert figure 5 again.
- Figure 6 formed of only five bar charts (A-E), while in result section authors had mention results about (fig 6 G&F)???
- Hepatic Stem Cells in the Models of T1D and T2D: (Fig. 6A-B) **NOT** (Fig. 6C-D) showed that the greatest number of OV6+ and KRT19+ cells in liver. Correct.

Discussion:

- Discussion section needs to be re-written in a better way and most of paragraphs need to be reconstructed.
- Correct the following words: an-imals, insu-lin, ex-pression, gen-eration, in-flammation, dia-betes, an-imals, gen-eration, insu-lin, in-crease, con-trol, re-storing, ex-tra).
- Remove the sentence: (In this study, for the first time, we demonstrated difference in the number of IPCs in the models of T1D and T2D.), since there is a previous study

investigated the same points of the current research https://doi.org/10.1016/j.scr.2020.101958

- Remove this repeated sentence in the 2nd paragraph in discussion (Glucose is the major physiologic regulator of insulin gene expression.) as it was mentioned before in the 4th paragraph in introduction section.
- Write the full name of the following abbreviations within the text (Glut, PNM,
- Correct the following sentence: So, hyperglycemia is a necessary, **but in**sufficient agent, **in stimulation of** hepatic insulin synthesis, **instead of** (bur unsufficient agent, stimulated hepatic insulin synthesis.).
