# Supplementary material for: Hepatic insulin synthesis increases in rat models of diabetes mellitus type 1 and 2 differently
Source: PLoS One. 2023 Nov 29;18(11):e0294432. doi: 10.1371/journal.pone.0294432 (PMC10686419; doi:10.1371/journal.pone.0294432)
Supplement: Attachment — Submitted filename: Response to Reviewers.docx [file pone.0294432.s003.docx]

*Dear colleagues,*

*We would like to thank you for your helpful comments. Please finf below a point-by-point response to your comments and questions.*

**Response to Academic Editor Comments:**

**Point 1:**

Please ensure that your manuscript meets PLOS ONE's style requirements, including those for file naming. The PLOS ONE style templates can be found at

https://journals.plos.org/plosone/s/file?id=wjVg/PLOSOne_formatting_sample_main_body.pdf and

<https://journals.plos.org/plosone/s/file?id=ba62/PLOSOne_formatting_sample_title_authors_affiliations.pdf>

**Response 1:** We ensure, that manuscript meets PLOS ONE's style requirements, including those for file naming.

**Point 2:**

Please note that funding information should not appear in any section or other areas of your manuscript. We will only publish funding information present in the Funding Statement section of the online submission form. Please remove any funding-related text from the manuscript.

**Response 2:**

We remove any funding-related text from the manuscript.

**Point 3:**

We note that the grant information you provided in the ‘Funding Information’ and ‘Financial Disclosure’ sections do not match.

When you resubmit, please ensure that you provide the correct grant numbers for the awards you received for your study in the ‘Funding Information’ section.

**Response 3:**

Done.

**Point 4:**

Thank you for stating the following financial disclosure: "122020900136-4 IIP UB RAS"

Please state what role the funders took in the study. If the funders had no role, please state: "The funders had no role in study design, data collection and analysis, decision to publish, or preparation of the manuscript."

If this statement is not correct you must amend it as needed.

Please include this amended Role of Funder statement in your cover letter; we will change the online submission form on your behalf.

**Response 4:**

Done.

**Point 5:**

Please expand the acronym “IIP UB RAS” (as indicated in your financial disclosure) so that it states the name of your funders in full.

This information should be included in your cover letter; we will change the online submission form on your behalf.

**Response 5:**

Done.

**Point 6:**

In your Data Availability statement, you have not specified where the minimal data set underlying the results described in your manuscript can be found. PLOS defines a study's minimal data set as the underlying data used to reach the conclusions drawn in the manuscript and any additional data required to replicate the reported study findings in their entirety. All PLOS journals require that the minimal data set be made fully available. For more information about our data policy, please see http://journals.plos.org/plosone/s/data-availability.

"Upon re-submitting your revised manuscript, please upload your study’s minimal underlying data set as either Supporting Information files or to a stable, public repository and include the relevant URLs, DOIs, or accession numbers within your revised cover letter. For a list of acceptable repositories, please see http://journals.plos.org/plosone/s/data-availability#loc-recommended-repositories. Any potentially identifying patient information must be fully anonymized.

Important: If there are ethical or legal restrictions to sharing your data publicly, please explain these restrictions in detail. Please see our guidelines for more information on what we consider unacceptable restrictions to publicly sharing data: http://journals.plos.org/plosone/s/data-availability#loc-unacceptable-data-access-restrictions. Note that it is not acceptable for the authors to be the sole named individuals responsible for ensuring data access.

We will update your Data Availability statement to reflect the information you provide in your cover letter.

**Response 6:**

Done. Minimal underlying data set was upload as a separate Supporting Information file.

**Point 7:**

Your ethics statement should only appear in the Methods section of your manuscript. If your ethics statement is written in any section besides the Methods, please move it to the Methods section and delete it from any other section. Please ensure that your ethics statement is included in your manuscript, as the ethics statement entered into the online submission form will not be published alongside your manuscript.

**Response 7:**

Done.

**Point 8:**

Please include a copy of Table 2 which you refer to in your text on page 24.

**Response 8:**

Done. We inserted Tables 1 and 2 immediately after the first paragraph in which they are cited.

**Point 9:**

Please upload a copy of Supporting Information Table 2 which you refer to in your text on page 14.

Done. We inserted Tables 1 and 2 immediately after the first paragraph in which they are cited.

**Response to Reviewer 1 Comments:**

**Point 1:**

General comments: Manuscript needs English language revising.

**Response 1:**

Done.

**Point 2:**

Paragraph should not start with abbreviation, so please write the full name, and here are some examples: IPS “third paragraph in introduction”, T1D “second paragraph in material and methods”, …ect.

**Response 2:**

Done.

**Point 3:**

Abstract: Line 6; Replace were not (done) with were not (performed).

**Response 3:**

Done.

**Point 4:**

Introduction: Correct the following words: per-manent to permanent, idi-opathic, proinsulin-, ex-pression, in-sulin, vec-tor, peculiar-ities, insu-lin, transdif-ferentiate, insu-lin, phago-, immunolog-ical, pit-cells, inflammation – in

**Response 4:**

Done.

**Point 5:**

Introduction is too long, there is no clear coherence and sequence in the writing, and most of the paragraphs require reconstruction and here are some examples for improvement:

Old one: In any case, the number of the functioning β-cells is insufficient in both T1D and T2D, and β-cells loss and dysfunction are the main reasons of hyperglycemia and associated with it complications [5].

New one: Loss and dysfunction of β-cells are the main reasons of hyperglycemia and its associated complications in both types of DM [5].

Old one: first, they are an additional source of insulin, the increase in the significance of which for the organism is logical to assume when pancreatic islets β-cells are damaged. Earlier we demonstrated [8] that in experimental T2D mass and functional activity of islet β-cells decrease faster, than of IPCs in pancreatic acini and ducts. Understanding the mechanisms and conditions, leading to the generation of IPCs in organs, are able to alleviate the problem of lack of β-cells. Second, since knowledge about ectopic insulin expression and, more, developmental plasticity of adult somatic cells and its reprogramming to committed lineages, unsufficient for complete and clear picture of this phenomenon, studying of extra-pancreatic insulin expression is also of theoretical interest.

New one: first, they are additional sources of insulin, as they showed significant increase within organism that are able to alleviate the problem when pancreatic islets β-cells are damaged as recorded in our previous study [8]. Second, to disclose the extra pancreatic insulin expression from adult somatic cells and reprogramming it to committed lineages.

**Response 5:**

We shortened and reconstructed the Introduction.

**Point 6:**

Introduction: Write the full name of the Pdx1 abbreviation within the text.

**Response 6:**

Done.

**Point 7:**

Introduction: Rewrite the aim of the study in a simple and clear form.

**Response 7:**

Done.

**Point 8:**

Material and methods: Correct the following words: ac-cording, fluo-rescent.

**Response 8:**

Done.

**Point 9:**

Material and methods: Insulin+, macrophages (F4/80+ cells), oval cells (OV6+ and KRT19+ cells), CD3+ and CD45+ cells were detected by IHC, (**replace** and **by** while) Pdx1+, MafA+, Ngn3+ cells were detected using fluorescent IHC.

**Response 9:**

Done.

**Point 10:**

Material and methods: Add data about the color and site of immune-expression (cytoplasmic or nuclear) in the stained cells.

**Response 10:**

Done. We specify the color and site of immune expression in the Figure Captions.

**Point 11:**

Material and methods: Morphometric analysis: specify the number of captured field from each slide/group.

**Response 11:**

The number of captured field was specified in the block “Morphometric analysis”: All morphometric evaluations were based on 20 microscopy fields per tissue section of each rat and were converted to the 1 mm2 of liver parenchyma (N/mm^2^).

**Point 12:**

RESULTS: Result section needs SUBSTANTIAL revision. Correct the following words: ob-served, insu-lin, De-pending, ana-lyzed, pro-tein, particu-larly, rear-rangement, regenera-tive

**Response 12:**

Done.

**Point 13:**

RESULTS: Write the full name of the following abbreviations within the text (AUC, HOMA-IR).

**Response 13:**

HOMA-IR – Done.

The full name of the abbreviation “AUC” there is in line # 313: The area under the curve (AUC) was calculated according to Sakaguchi et al. (2016) using the trapezoidal approximation of PG levels [38].

**Point 14:**

RESULTS: Figure (1 C) compares ND and T2D, while figure legend compares T2D from T1D, revise it.

**Response 14:**

Done.

**Point 15:**

RESULTS: Results of (Figure 1 from F to J) are not mentioned in figure legend, need to be added.

**Response 15:**

Done.

**Point 16:**

RESULTS: Results of (Figure 2 A, C to I) are not mentioned in the text, need to be added.

**Response 16:**

Done.

**Point 17:**

RESULTS: Add scale bar in all photomicrographs in figure 2.

**Response 17:**

Done.

**Point 18:**

RESULTS: Reorganize the writing of result section (text and legend) according to figures distribution. For example authors write about figure 1 A-D, then fig 2B, then figure 6, then return to figure 1F-J and so on…...

**Response 18:**

Done.

**Point 19:**

RESULTS: For scientific benefit, it is better to insert each bar charts immediately below their corresponding histological or IHC figures.

**Response 19:**

Done.

**Point 20:**

RESULTS: Figures 5 and 6 are the same figure???? Need to insert figure 5 again.

**Response 20:**

Done.

**Point 21:**

RESULTS: Figure 6 formed of only five bar charts (A-E), while in result section authors had mention results about (fig 6 G&F)???

**Response 21:**

Done. All figures were remaked.

**Point 22:**

RESULTS: Hepatic Stem Cells in the Models of T1D and T2D: (Fig. 6A-B) NOT (Fig. 6C-D) showed that the greatest number of OV6+ and KRT19+ cells in liver. Correct.

**Response 22:**

We fixed in Figure Caption.

**Point 23:**

Discussion: Discussion section needs to be re-written in a better way and most of paragraphs need to be reconstructed.

**Response 23:**

Done.

**Point 24:**

Discussion: Correct the following words: an-imals, insu-lin, ex-pression, gen-eration, in-flammation, dia-betes, an-imals, gen-eration, insu-lin, in-crease, con-trol, re-storing, ex-tra).

**Response 24:**

Done.

**Point 25:**

Discussion: Remove the sentence: (In this study, for the first time, we demonstrated difference in the number of IPCs in the models of T1D and T2D.), since there is a previous study investigated the same points of the current research <https://doi.org/10.1016/j.scr.2020.101958>

**Response 25:**

The <https://doi.org/10.1016/j.scr.2020.101958> research is the first study have demonstrated that functional insulin-producing cells (IPCs) can be generated by using induced pluripotent stem cells (iPSCs) derived from as well non-diabetic (ND) as T1D and T2D individuals in vitro.

In our research we evaluate the mass of IPCs in the liver in the rat models of T1D and T2D with the comparable glycemic level in vivo and try to evaluate factors impact emerging of IPCS in the liver.

**Point 26:**

Discussion: Remove this repeated sentence in the 2nd paragraph in discussion (Glucose is the major physiologic regulator of insulin gene expression.) as it was mentioned before in the 4th paragraph in introduction section.

**Response 26:**

Done.

**Point 27:**

Discussion: Write the full name of the following abbreviations within the text (Glut, PNM).

**Response 27:**

Done.

**Point 28:**

Discussion: Correct the following sentence: So, hyperglycemia is a necessary, but insufficient agent, in stimulation of hepatic insulin synthesis, instead of (bur unsufficient agent, stimulated hepatic insulin synthesis.).

**Response 28:**

Done.

**Response to Reviewer 2 Comments:**

**Point 1** **(Major):** E,F,G in Figure.2 was too hard to see or calculate the positive cells. The authors should replace the new or visuable pictures.

**Response 1:**

Done.

**Point 2 (Minor):**

There were some mistake letter such as "insu-lin-producing cells" and "immunolog-ical reactions". The authors should caefully revise these letter in the manuscript. Also, English grammer should be revised.

**Response 2:**

Done.

**List of changes**

1) We revised the numbering of affiliations. The right variant is:

**Musa Abidov ^1^, Ksenia Sokolova ^2^, Irina Danilova ^2^, Madina Baykenova ^3^, Irina Gette ^2^, Elena Mychlynina ^2^, Burcin Aydin Ozgur ^4,5*^ , Ali Osman Gurol ^6,7^, M. Temel Yilmaz ^8^**

^1^ Institute of Immunopathology and Preventive Medicine, Ljubljana, Slovenia

^2^ Institute of Immunology and Physiology, Ural Branch of the Russian Academy of Sciences, Yekaterinburg, Russian Federation

^3^ Kostanay Oblast Tuberculosis Dispensary, Kostanay, Republic of Kazakhstan

^4^ Department of Medical Biology and Genetics, Faculty of Medicine, Demiroglu Bilim University, Istanbul, Turkey

^5^ Diabetes Application and Research Center, Demiroglu Bilim University, Istanbul, Turkey

^6^ Department of Immunology, Aziz Sancar Institute of Experimental Medicine, Istanbul University, Istanbul, Turkey

^7^ Diabetes Application and Research Center, Istanbul University, Istanbul, Turkey

^8^ International Diabetes Center, Acibadem University, Istanbul, Turkey

2) We remove any funding-related text from the manuscript.

We expand the acronym “IIP UB RAS”.

In the Funding Statement section of the online submission form one’s should to note the follow:

**“**This research was carried out within the state assignment of the Institute Immunology and Physiology Ural Branch Russian Academy of Science (IIP UB RAS) № 122020900136-4. The funders had no role in study design, data collection and analysis, decision to publish, or preparation of the manuscript. The work was performed using the equipment of the Shared Research Center of Scientific Equipment of the IIP UB RAS.”
