# Supplementary material for: Hepatic insulin synthesis increases in rat models of diabetes mellitus type 1 and 2 differently
Source: PLoS One. 2023 Nov 29;18(11):e0294432. doi: 10.1371/journal.pone.0294432 (PMC10686419; doi:10.1371/journal.pone.0294432)
Supplement: Attachment — Submitted filename: Response to Reviewers.docx [file pone.0294432.s005.docx]

*Dear colleagues,*

*We would like to thank you for your helpful comments. Please finf below a point-by-point response to your comments and questions.*

**Response to Reviewer #1 Comments:**

**Point 1:**

Introduction:

**Add reference to this paragraph** -

«Extra-islet and extra-pancreatic IPCs are the spotlight of researchers for at least two reasons: first, they are additional sources of insulin and probably are able to alleviate hyperglycemia and prevent complications of diabetes. Secondly, ectopic insulin expression in functional mature cell in different tissues is of fundamental theoretical interest and expand the understanding of tissue adaptation in pathology condition» (Reference???).

**Response 1:**

Done.

**Point 2:**

RESULTS

**Remove number 38 from this paragraph**:

«Rats with T2D showed poor glucose tolerance, as evident by higher AUC value observed in T2D group compared to ND group (p < 0.05) (Fig. 1С) [38])».

**Response 2:**

Done.

Removed reference was the article of Sakaguchi, K. et al. *Glucose area under the curve during oral glucose tolerance test as an index of glucose intolerance. Diabetol Int 2016, 7(1), 53–58,* which discuss the usage of an oral glucose tolerance test (OGTT) for clear detection of glucose intolerance in the early stage of diabetes.

**Point 3:**

**Figure 2 & 5 legends: Cite the group according to figure letters (A, B &C):**

(A) ND – non-diabetic group, (B) T1D – type 1 diabetes, (C) T2D – type 2 diabetes.

**Response 3:**

Done.

**Point 4:**

**Reorganize the text to be matching with figure order:**

**Markers of liver injury and inflammation in blood**

To evaluate functional state of hepatocytes in the models of T1D and T2D, we analyzed

activity of aminotransferases (**AST and ALT**) **instead of** (ALT and AST), alkaline phosphatase (**ALP**) **instead of** (AP) and protein content in blood plasma (Fig. 3A-E). **There was an increase in serum AST level in T1D, compared to ND. In contrast, AST level in T2D did not change**. ALT is a rather specific marker of hepatocellular injury [45]. Increase in ALT level compared to normal was higher in T2D group than in T1D. A high ALT level led to a decrease in AST/ALT ratio (De Ritis coefficient) in T2D. **((((There was an increase in serum AST level in T1D, compared to ND. In contrast, AST level in T2D did not change)))) REMOVE the sentence.**

**Response 4:**

Done.

**Point 5:** Add letter (E) to figure 3

**Response 5:**

Done.
